# Ethno-evolutionary insights and missed critical periods hypothesis of Autism Spectrum Condition

**DOI:** 10.3389/frcha.2026.1838798

**Published:** 2026-07-13

**Authors:** Costanza Colombi, Martin Brüne

**Affiliations:** 1 Stella Maris Foundation (IRCCS), Calambrone, Italy; 2 Ruhr-Universitat Bochum, Bochum, Germany

**Keywords:** autism (ASC), critical period, hypothesis, infants, preemptive intervention

## Abstract

This article proposes an ethno-evolutionary “missed critical periods” hypothesis of Autism Spectrum Condition (ASC), integrating developmental neuroscience with anthropological and ethological perspectives. Drawing on evidence that the first year of life is characterized by heightened brain plasticity and rapidly narrowing sensory–social attunement, the manuscript argues that core ASC symptoms reflect the downstream outcome of early hyper-focalized attentional styles interacting with historically situated caregiving ecologies, rather than a fixed, context-independent defect. Early prospective studies of high-likelihood infants show that subtle alterations in gaze, socio-communicative reciprocity and restricted, repetitive behaviors emerge within the first months, opening a window for pre-emptive, parent-mediated intervention. Converging data from pre-emptive interventions suggest that targeting infant–caregiver micro-interactions during this window can substantially modify developmental trajectories and, in some cases, reduce or mitigate disability. Placing these findings in an evolutionary frame, the article hypothesizes that genetic liabilities for ASC have long been present across human groups, but that dense, multi-caregiver, high-contact rearing practices typical of hunter–gatherer societies may have buffered against the manifestation of disabling phenotypes. The authors highlight implications for contemporary Western child-rearing norms and outline future research directions, including randomized pre-emptive trials with neurophysiological outcomes and cross-cultural studies in traditional societies.

## Introduction

What is currently known as Autism Spectrum Condition (ASC) refers to a conglomerate of psychiatric conditions that appeared in the scientific literature quite recently. Austrian psychiatrist Leo Kanner published a seminal paper in 1943 describing infantile autism as a specific and distinct clinical condition and hypothesizing a neurobiological etiology (1). Kanner reported 11 children identifying two areas of symptomatology as characteristic of their condition including, 1. Severe difficulties in social interaction and relationship, 2. Resistance to change and insistence in sameness and repetition. Similarly, Hans Asperger's work on “autistic psychopathy” concerned a disorder where stereotyped behavior and disturbances of social interaction were not accompanied by intellectual impairment (2).

While the above-mentioned characteristics of ASC are still present in the diagnostic criteria of the DSM-5-R (3) and ICD-11 (4), a plethora of etiological hypotheses and theories have been proposed during the last 80 year in various areas of human mental health science including psychodynamic, socio-cognition, neurobiology, and genetics. Within a psychodynamic context, Bruno Bettelheim proposed the theory of the “refrigerator mother” as causative of the ASC condition (5), which is now considered obsolete and unequivocally refuted by the clinical and scientific community. In the realm of socio-cognition sciences several explanatory accounts have been proposed such as the Central Coherence Theory (6), the Theory of Mind Theory (7), and the Social Motivation Theory (8). At a neurobiological level, a disruption in the mirror neuron mechanisms has been suggested (9), as well as dysfunctional synaptic pruning during early stages of brain development (10). Some well identified patterns of ASC suggest a clear genetic component. For instance, ASC is more frequent in male in comparison to female individuals (11). Moreover, once an autistic child is present in a family, the risk for a subsequent sibling to carry the ASC condition as well raises to approximately 20% in comparison to the 2%–3% incidence in the general population (12). Nevertheless, a specific and universally identifiable genotype has not yet been found in autistic individuals (13), and evidence is mounting that there is genetic heterogeneity involving multiple loci in ASC (14). Beyond familiarity patterns, additional factors are correlated with ASC including prematurity at birth, Small for Gestational Age (SGA) body weight, perinatal complications, parental age, and environmental pollution (15). Because we sympathize with the view that ASC is a neurodivergent condition that can lead to disability and impairment, but not necessarily so, in the manuscript we refer to it with the term Autism Spectrum Condition (ASC) rather than Autism Spectrum Disorder (ASD). Importantly, throughout this paper we distinguish between three overlapping but distinct constructs: (a) ASC as a neurodevelopmental condition characterized by a specific cognitive-attentional profile; (b) sub-threshold autistic traits present in the general population and linked to common genetic variants; and (c) disability arising from the mismatch between a neurodivergent profile and a given social or physical environment. Our preventive framing targets disability and impairment, not the neurodivergent profile itself, consistent with recent theoretical reformulations of autism as an emergent transactional phenomenon shaped by the quality of early caregiving experience (16).

## ASC prodromes during the first year of life

During the last 20 years, early recognition of ASC has made important progress. Since the early 2000s, several multi-centric studies have followed up the development of children with an autistic sibling since birth, given their high risk to express ASC as well (17, 18). Infants who will receive an ASC diagnosis show signs in the area of restricted and repetitive behaviors, including prolonged visual inspection of objects, insistence in the repetitive use of objects, and repetitive atypical movements usually related to their hands (15). Additionally, signs are observed in the socio-communication area and include progressive decline in eye contact starting at 2–6 months of age, limited response to others' social initiation, limited or absent social smile which usually emerges at around 6 weeks of age in neurotypical development, difficulty in sustaining socio-communicative reciprocity, limited or absent orientation and initiation toward social stimuli (17). Moreover, nonspecific signs of a possible neurodevelopmental disorder are often present such as motor immaturity, scarce of absent vocalization, or feeding difficulties (19). Regarding temperament, most infants with a future ASC demonstrate passivity and diminished activity during the first months of life (20). On the other hand, some extremely irritable and very difficult to suit infants, in the absence of a physical condition, have also been described (21).

Early recognition of ASC is, however, most helpful if we can propose an intervention. Indeed, mere identification may even increase parental concerns without offering an opportunity for intervention. Despite being in its infancy, parent-mediated intervention for infants with ASC prodromes is producing surprising results. The numerically limited but rigorous studies indicate a clear reduction of the disability and suggest a possible prevention of the diagnosis in at least some children (22, 23). The key of these outstanding outcomes is the precocity of the intervention delivered during the first year of life when cerebral plasticity is high (17). In the clinical and research world, we start to pave the concrete possibility of a massive impact on developmental trajectories of infants with ASC prodromes when the intervention begins before 12–15 months of age (22, 23). An additional strength of this innovative clinical research field pertains to the low amount of time spent by the therapist in interaction with the family to reach results not even thinkable until recently. The studies show very promising results with interventions lasting approximately 6 months with one 1 h therapist—family session per week (20, 21), while the usual intervention for an older child not identified early in development requires even 20 or more hours per week in combination with a special education teacher present for the whole time of school attendance (24, 25).

A notable exception concerns syndromic forms of ASC with identifiable monogenic or chromosomal etiologies. Approximately 10%–15% of individuals with autism spectrum disorder (ASD) have a clearly identifiable monogenic or chromosomal condition, often associated with syndromic presentations and a high rate of intellectual disability (e.g., fragile X syndrome, tuberous sclerosis complex, Rett syndrome (26, 27). In this subgroup, the causal role of specific genetic variants is predominant, and the ethnoevolutionary hypothesis of “missed critical periods” and its implications for primary prevention are not directly applicable to these forms of syndromic ASD, which are better conceptualized within a framework of highly penetrant genetic etiologies (24). Nevertheless, converging clinical guidelines and consensus papers indicate that early, developmentally informed and family-centered intervention remains recommended for all autistic children—regardless of etiology—to support developmental functioning, adaptive behavior and quality of life, rather than to “prevent” the onset of the condition (28, 29).

## Evolutionary psychiatry

Evolutionary psychiatry is a discipline that tries to explain mental health disorders in evolutionary terms (30). The focus of evolutionary psychiatry is to provide scientific explanations per psychiatric disorders rather than treatments. A key principle of such approach is evolutionary mismatch, a mechanism explaining psychopathology as caused by modern environment. Evolutionary psychiatry attempts to integrate traditional human mental health science such as social psychology, behaviorism, psychoanalysis within a holistic discipline relate to evolutionary biology (28).

## Ethno-evolutionary considerations and missed critical periods of autism spectrum condition

The following section presents a partly speculative, hypothesis-generating framework rather than a systematic review; empirical claims are clearly distinguished from theoretical propositions throughout. The incidence of ASC seems to suggest an exponential increase, at least in Western cultures. For example, in the United States the Center for Diseases and Control (CDC) has reported a dramatic change of incidence rates from 1:150 in 2000 to 1:31 in 2023 (31). This rise in numbers of ASC may be related to improved recognition, in combination with broadened diagnostic criteria, including individuals without cognitive or verbal disabilities. However, these factors do not seem to completely explain the exponential increase in number of cases.

A speculative hypothesis suggests that some characteristics of ASC are associated with greater success in specific work environment that emerged only since the industrial revolution. It is well known that approximately 10% of physicists, mathematicians and programmers present with characteristics of ASC; however, there is no indication that possessing aspects of ASC is associated with greater than average reproductive success (i.e., greater number of offspring) that could potentially account for the rising numbers of individuals diagnosed with ASC, even though the rapidity of suspected increment in prevalence of genes associated with ASC is largely at odds with evolutionary timing, when, for instance, compared with changes in skin pigmentation in Europe over the last 40 thousand years (32), which happened at a much slower pace.

Nevertheless, considering evolutionary timing it seems plausible to assume that gene patterns correlated with ASC were present many thousands of years ago, and should be present in present-day hunter gatherer populations. Indeed, Hunt and Jaeggi (33, 34) have argued that genes predisposing to a broader autistic phenotype should be present in every human population, because they may either be associated with biological fitness advantages, if an individual carrying these traits finds a niche within the tribe or band, where the benefits (e.g., systemizing abilities or general intelligence) outweigh the costs (e.g., low sociosexuality). Moreover, these authors estimate that, if a broadly defined autistic phenotype existed, it should be present at a prevalence rate of about 7 percent, suggesting that at least one or two individuals in every tribal society should be affected (31, 32). However, even when taking into account the possibility of assortative mating, i.e., mating of individuals expressing similar personality traits, the evidence is sparse, if not absent, that in traditional (hunter-gatherer) societies, individuals with a typical autistic spectrum phenotype can been found.

So, could this mean that disabling autistic phenotypes were less prevalent or expressed in attenuated form in hunter-gatherer societies, due to the specific ways of socialization in tribal societies, despite genes predisposing to the condition being present? An ethological perspective on the actual behavior of autistic individuals may shed light on this question. More than 50 years ago, Nobel laureate Nicolaas Tinbergen found the topic to be of such relevance that he devoted half of his Nobel laureate speech to therapeutic approaches to autism, based on ethological observation (35). In fact, Tinbergen noticed that children diagnosed with autism showed “an extremely rich set of expressions that are all correlated with overt avoidance” (of social contact). Importantly, Tinbergen (35) emphasized that none of the behavioral expressions found in autistic children were specific to the clinical condition. Instead, he posited that all elements were part of the typical repertoire of children, which, however, usually occurred in situations associated with motivational conflict. The ethological term for such behaviors is “displacement activities”. Displacement activities are usually characterized by locomotor movements or self-grooming elements. A typical situation where displacement activity occurred is in a motivational conflict between fear and exploratory behavior, as Tinbergen pointed out. This is highly important, because the ethological perspective suggests that elements of behavior found in autistic children are responsive to the (social) environment. Another issue raised by Tinbergen in his Nobel laureate speech was that parental behavior could be of the essence. He speculated that parents of autistic children could be inexperienced or overly apprehensive, or perhaps even stressed. From our point of view, this is an extremely important hypothesis, because it could explain why autistic behavior is barely seen in children in hunter-gatherer societies. Indeed, one important ethnographic observation in hunter-gatherer societies is that infants ALWAYS grow up in groups of children, where the older children care for the younger, carry them around and introduce them to the environment (36). It could therefore be that the specific ways of child rearing in hunter-gatherers have a strong preventive effect on the development of autism (and other psychopathological conditions). After acknowledging Tinbergen's ethological sensibility and his attention to observable interaction patterns, it is important to clearly distance the present proposal from his etiological assumptions. In Tinbergen's work, the focus on parental behavior slid toward a de facto causal model in which specific maternal and familial styles were implicitly treated as primary determinants of autistic pathology. From an ethnoevolutionary standpoint, this move is problematic on at least two levels: empirically, because current evidence supports a multifactorial neurodevelopmental aetiology of autism rather than a quasi-monocausal parental one; and conceptually, because it narrows the field of explanation to the dyad, neglecting the broader cultural ecologies of caregiving in which both parents and infants are embedded. Consistent with this, recent observational data from WEIRD samples show that parenting style correlates with autistic traits in toddlers even in the absence of a clinical intervention, suggesting that caregiving quality modulates the expression of neurodivergent profiles within typical Western environments (37).

The causal mechanism linking parental responsiveness to phenotypic outcome has been independently elucidated through mediation analyses of the PACT program, which identified a two-step pathway from increased parental synchrony to improved child social initiation, to sustained reduction in autism symptom severity over six years of follow-up (16). In this article, while appreciating Tinbergen's observational acuity, ASC is not read as an effect of “wrong parenting”, but as a possible developmental outcome of the encounter between specific neurocognitive profiles and historically situated environments of care, interaction and classification.

## Ethno-evolutionary and missed critical periods hypothesis

Accordingly, our ethno-evolutionary “missed critical period” hypothesis proposed here lays its foundation on the principles of neurobiology, including brain plasticity, critical periods, epigenetics, ethology, and anthropology. Brain plasticity is inversely related to the individual age and it is at its maximum potential during the first year of life (38). Plasticity means fast acquisition of competences such as language in combination with loss of skills not used through synaptic pruning (38). For instance, infants are able to distinguish all phonemes of all languages of the world until approximately 4–6 months of age (39). Subsequently, phoneme distinction progressively fades and it is fully maintained only for the languages we are exposed to (39).

Going back to ASC, a clear common sign across all autistic individuals is poor eye contact (40). Poor eye contact is observable in young children, adults, individuals with cognitive and verbal disabilities and individuals with cognitive and verbal abilities well above average. Commonly, ASC infant researchers used to hypothesize that limited or absent eye contact was observable since birth in individuals with a future ASC. In reality, as it has happened for many ASC hypotheses, data are counterintuitive. Through the infant sibling design (41), with the use of eye-tracking technology it has been shown that not only children with a future ASC establish eye contact at birth, they even look at eyes for a higher amount of time in comparison to their neurotypical peers during the first 2–6 months of life (42). After this hyper eye-looking period of time, infants with a future ASC show a continuous decline in eye contact starting at 2–6 months of age (42), while in their neurotypical peers, the use of eye contact gets more and more sophisticates. At 6 months infants with a future ASC look more at the mouth region in comparison to their typical peers (43). We hypothesize that the symptoms described in the current diagnostic classifications of ASC, that is, in the area of socio-communication and in the area of repetitive and restricted interest, are not the origin of the condition but outcomes of a peculiar cognitive attentional style characterized by hyper-focalization that can lead to disability during the epigenetic process of gene—environment interaction. Perhaps ASC infants at birth are prone to establish eye contact similarly to their neurotypical peers, but they lack in flexibility and attention shifting. Hyper-focalization could lead the infant to initially attend without flexibility and scarcity of shifting at first to the eyes, then to the mouth and finally mainly to the world of objects, in comparison to the social world once motor abilities improve and the infant spend less and less time close to caregivers. Similarly to the loss of phoneme distinction at around 4–6 months of age, ASC infants could perhaps lose the ability to modulate eye contact if not intensively practiced during the first year of life. Such a process could explain other losses, lack of acquisitions or not fluid acquisitions that characterize the development of ASC children, including language, gestures, motor abilities, cognitive skills, social cognition.

We hypothesize that in human hunter-gatherer societies, which characterize approximately 95% of human evolutionary history, the presence of individuals with hyper-focalization and attention to detail could have been advantageous for tasks such as hunting and food gathering. In contrast to contemporary WEIRD settings, these societies are organized in small, highly prosocial residential bands, where adults and children live in dense networks of exchange and mutual support rather than in isolated nuclear households. Infants and young children typically receive continuous physical contact and care from multiple caregivers, including parents, older siblings and other alloparental figures, creating a high-contact, multi-caregiver ecology. Ethnographic work suggests that 40%–50% of direct childcare in many foraging groups is provided by alloparents, a pattern associated with secure attachment and reduced risk of abuse and neglect (36, 44). Within this ethnoevolutionary frame, such intense, distributed nurturing during the first year of life—when brain plasticity and socio-emotional critical periods are at their peak—may have buffered the developmental impact of ASC-related hyper-focalized cognitive styles, preventing or attenuating the crystallization of disabling phenotypes despite similar underlying genetic liabilities (33, 34, 38, 45).

Over the last decade, several pre-emptive, parent-mediated programs have demonstrated that it is possible to influence the developmental course of infants who show prodromal signs of Autism Spectrum Condition (ASC) during the first year of life. Pilot work on Infant Start (a downward extension of the Early Start Denver Model) and the randomized controlled trial of the iBASIS–Video Interaction to Promote Positive Parenting (iBASIS-VIPP) indicate that brief, low-intensity interventions focused on coaching caregivers to finely attune to the infant's signals can reduce the severity of socio-communicative symptoms and restricted/repetitive behaviors, and in a subset of infants are associated with the absence of a clinical ASC diagnosis at preschool age (22, 23). In a similar vein, the FIRRST (Fostering Infant Responsivity and Reciprocity—Support to Thrive) approach has shown, in case-based and preliminary data, marked reductions in early ASC symptomatology—such as limited social initiation/reciprocity, hyper-focalized attention patterns and developmental immaturity—together with acceleration of developmental trajectories into the typical range when intervention begins in the first year of life (46). Green (2022) provides the theoretical synthesis of this intervention evidence base within a transactional model of autism development, arguing that the autistic phenotype is emergent from and malleable by the quality of early interpersonal experience (16). Rather than attempting to “normalize” an atypical neurocognitive style, these interventions operate by reshaping the caregiving ecology at a time of heightened brain plasticity, intensifying contingent, flexible, and responsive exchanges so as to prevent the consolidation of disabling developmental pathways while respecting neurodivergent tendencies (24, 38).

## Limitations

Several important limitations of the present framework must be acknowledged. First, the claim that disabling ASC phenotypes are attenuated in hunter-gatherer societies rests primarily on negative evidence — the absence of documented cases — which may reflect diagnostic invisibility, different cultural classification of atypical behaviour, or survival bias rather than genuine phenotypic attenuation [but see Melamu et al., 2024 (47) for a recent anecdotal description of ASD in the Setswana culture from South Africa]. Second, the causal direction between caregiving ecology and neurodevelopmental outcomes cannot be established from existing ethnographic or intervention data; observed correlations between high-contact rearing and favorable developmental trajectories may partly reflect shared genetic factors (gene-environment correlation) rather than a purely environmental effect. Third, the applicability of findings from pre-emptive interventions in high-income WEIRD settings to hunter-gatherer or low- and middle-income contexts remains to be demonstrated. Fourth, the current hypothesis focuses on non-syndromic ASC; as noted above, its implications do not extend to syndromic forms with highly penetrant monogenic or chromosomal aetiologies. Future empirical work — including ethnographic studies in small-scale societies and adequately powered cross-cultural trials — is needed to test these propositions rigorously. This measured position is consistent with Green's observation that phenotypic malleability demonstrated in intervention trials is real but bounded — most children remain neurodivergent after pre-emptive intervention (16) — and our hypothesis makes no stronger claim.

## Future directions

Future directions in this field include, first, adequately powered randomized controlled trials of pre-emptive, parent-mediated interventions initiated within the first year of life, coupled with long-term follow-up to school age and beyond. Such studies should systematically integrate neurophysiological and neuroimaging markers (e.g., EEG/ERP, eye-tracking, fNIRS) to clarify how changes in infant–caregiver micro-interactions translate into alterations of neural circuitry during critical periods of plasticity (24, 38, 40). Initial proof-of-concept data from the FIRRST program, delivered as a telehealth, parent-mediated naturalistic developmental behavioral intervention for infants under 15 months, lend empirical support to the feasibility and preliminary efficacy of this pre-emptive model and directly justify a multicenter randomized trial (48).

Second, research should extend beyond WEIRD populations to examine whether similar pre-emptive strategies are feasible, acceptable, and effective within diverse cultural ecologies of caregiving, including low- and middle-income countries and traditional societies. Ethnographic and mixed-methods studies in hunter–gatherer and other small-scale societies could be particularly informative in testing the hypothesis that dense, multi-caregiver, high-contact rearing environments mitigate the expression of disabling ASC phenotypes despite comparable genetic liability (34, 35, 46).

Third, future work should identify which infants are most likely to benefit from pre-emptive approaches, using multimodal risk stratification that combines familial risk, fine-grained behavioral markers, and biological signatures. This includes delineating individual differences in treatment response and “for whom and under what conditions” early interventions such as Infant Start, iBASIS-VIPP and FIRRST are most effective, with the dual aim of preventing disability while respecting neurodivergent trajectories (22, 23, 25, 46, 48).

We hypothesize that intervention during the first year of life may reduce and/or mitigate the disability associated with ASC. We further hypothesize that in hunter–gatherer societies ASC symptomatology, if present at all, may be attenuated and less likely to lead to disabling cognitive and verbal difficulties. If confirmed, these hypotheses would support the need for a critical reconsideration of rearing practices commonly adopted in Western, industrialized, high-income societies during the first years of life.

## Data Availability

Publicly available datasets were analyzed in this study. This data can be found here: This is a Perspective Article. Data are not presented.
